# Comparative Profiling of Metastatic 4T1- vs. Non-metastatic Py230-Based Mammary Tumors in an Intraductal Model for Triple-Negative Breast Cancer

**DOI:** 10.3389/fimmu.2019.02928

**Published:** 2019-12-17

**Authors:** Jonas Steenbrugge, Niels Vander Elst, Kristel Demeyere, Olivier De Wever, Niek N. Sanders, Wim Van Den Broeck, Luc Dirix, Steven Van Laere, Evelyne Meyer

**Affiliations:** ^1^Laboratory of Biochemistry, Department of Pharmacology, Toxicology and Biochemistry, Faculty of Veterinary Medicine, Ghent University, Merelbeke, Belgium; ^2^Translational Cancer Research Unit Antwerp, Center for Oncological Research, General Hospital Sint-Augustinus, Wilrijk, Belgium; ^3^Cancer Research Institute Ghent, Ghent, Belgium; ^4^Laboratory of Experimental Cancer Research, Department of Human Structure and Repair, Ghent University, Ghent, Belgium; ^5^Laboratory of Gene Therapy, Department of Nutrition, Genetics and Ethology, Faculty of Veterinary Medicine, Ghent University, Merelbeke, Belgium; ^6^Department of Morphology, Faculty of Veterinary Medicine, Ghent University, Merelbeke, Belgium

**Keywords:** triple-negative breast cancer, intraductal model, 4T1 mammary tumor cells, Py230 mammary tumor cells, tumor immunology

## Abstract

The transition of ductal carcinoma *in situ* (DCIS) to invasive carcinoma (IC) in breast cancer can be faithfully reproduced by the intraductal mouse model. Envisaging to use this model for therapeutic testing, we aimed to in-depth characterize the tumor immunity associated with the differential progression of two types of intraductal tumors. More specifically, we focused on triple-negative breast cancer (TNBC) and intraductally inoculated luciferase-expressing metastatic 4T1 and locally invasive Py230 cells in lactating mammary glands of syngeneic BALB/c and C57BL/6 female mice, respectively. Although the aggressive 4T1 cells rapidly formed solid tumors, Py230 tumors eventually grew to a similar size through enhanced proliferation. Yet, ductal tumor cell breakthrough and metastasis occurred earlier in the 4T1- compared to the Py230-based intraductal model and was associated with high expression of matrix metalloproteinase (MMP)-9, vascular endothelial growth factor (VEGF), chitinase 3-like 1 (CHI3L1) and lipocalin 2 (LCN2) as well as an increased influx of immune cells (mainly macrophages, neutrophils and T-cells). Moreover, activated cytotoxic T-cells, B-cells and programmed death-1 (PD-1)-positive cells were more prominent in the 4T1-based intraductal model in line with enhanced pro-inflammatory cytokine and gene expression profiles. Py230-based tumors showed a more immunosuppressed anti-inflammatory profile with a high amount of regulatory T-cells, which may account for the decreased T-cell activation but increased proliferation compared to the 4T1-based tumors. Taken together, our results highlight the differential immunological aspects of aggressive metastatic and non-aggressive intraductal progression of 4T1- vs. Py230-based tumors, providing a base for future studies to explore therapy using these intraductal TNBC models.

## Introduction

Breast cancer is the most frequently diagnosed and deadly cancer in women (1). At the initial stage, breast tumors typically remain inside the mammary ducts (i.e., ductal carcinoma *in situ*, DCIS), but then may quickly become invasive in the surrounding fat tissue and metastasize to distant organs. Although the immune system is primed to attack tumor cells during this disease process, it mostly fails to stop the overall breast tumor progression. It stands for reason that immunotherapy, that aims at restoring the anti-tumor immune response, may be effective in quenching breast cancer (2). Mouse models that replicate human breast cancer play an essential role in the preclinical screening of such drugs. To this end, we and others have shown that inoculation of mammary tumor cells in the murine mammary ductal environment, also referred to as the mouse intraductal (MIND) model, provides a significant advantage over the classical fat pad model as it allows to monitor disease progression from early DCIS to invasive carcinoma (IC) (3–10). Recently, we at first identified the stimulatory role of macrophage polarization in ductal breakthrough and metastatic progression in triple-negative breast cancer (TNBC) using a model based on intraductal inoculation of 4T1 mammary tumor cells in syngeneic BALB/c mice, suggesting that macrophage polarization is a potential immunotherapeutic target (4). Nevertheless, a more thorough knowledge of tumor immunological processes during the *in situ*, invasive and metastatic phases of breast cancer development is warranted to further validate this innovative intraductal model as a screening tool for therapy.

In the current study, we characterized the immune landscape within the intraductal model, focusing on TNBC. More specifically, we intraductally inoculated and compared the growth and tumor immunology of the aggressive BALB/c-derived 4T1 cell line vs. the slowly growing C57BL/6-derived Py230 cell line both in a syngeneic background. We here identify that Py230 tumor cells are able to catch up with 4T1 tumors by 6 weeks (w) post-inoculation (p.i.) through an enhanced cellular proliferation, but show no systemic advancement at that time. As tumor cells invaded through the ductal barrier, the tumor microenvironment significantly changed and showed increased immune cell influx. The different tumor outgrowth was also associated with a differential tumor immunology as 4T1 primary tumors were more pro-inflammatory showing high amounts of activated cytotoxic T-cells and immune-stimulating cytokines, and Py230 primary tumors were more anti-inflammatory containing a high number of regulatory T-cells, weak cytotoxic T-cell activation, and increased immunosuppressive cytokines. Taken together, the 4T1- and Py230-based intraductal model represent a different type of TNBC outgrowth and immune microenvironment that can be explored as preclinical tool for testing therapeutic strategies.

## Materials and Methods

### Mice

Wild-type BALB/c and C57BL/6 mice were purchased from Envigo. Albino NF-κB luc/Tyr^−/−^ C57BL/6 mice, which carry a heterozygous nuclear factor-kappa B (NF-κB) luc transgene and lack the tyrosinase-expressing gene responsible for coat color, were a kind gift from Harald Carlsen (11). No genetic drift was observed during the period of the study based on a 384 single nucleotide polymorphism (SNP) panel performed by Charles River Genetic Testing Services. Animals were conventionally housed with access to food and water *ad libitum*. All animal research was conducted in accordance with the recommendations in the Guide for the Care and Use of Laboratory Animals of the National Institutes of Health and approved by the Committee on the Ethics of Animal Experiments of The Faculty of Veterinary Medicine at Ghent University (approval numbers: EC2015/127, EC2016/56, and EC2017/80).

### 4T1 and Py230 Cell Culture

BALB/c-derived 4T1 mammary tumor cells constitutively expressing firefly luciferase were a kind gift from Prof. Clare Isacke (Breakthrough Breast Cancer Research Centre, London, UK). These cells resemble the aggressive metastatic characteristics of human TNBC [lacking expression of estrogen receptor (ER), progesterone receptor (PR) and human epidermal growth factor receptor 2 (HER2)] (12, 13). Py230 mammary tumor cells were purchased from the American Type Culture Collection (ATCC). These cells were originally derived from spontaneous tumors in MMTV-PyMT transgenic mice with a C57BL/6 background and have been used to model TNBC (14). Cell lines were maintained at 37°C and 5% CO_2_ in Dulbecco's Modified Eagle's Medium (DMEM) supplemented with 10% heat-inactivated fetal bovine serum (FBS), 100 U/ml penicillin and 100 μg/ml streptomycin (Thermo Fisher Scientific, Waltham, MA, USA) for 4T1 cells and Ham's F-12K (Kaighn's) Medium (Thermo Fisher Scientific) supplemented with 0.1% MITO+ Serum Extender (Corning), 5% heat-inactivated FBS, 100 U/ml penicillin and 100 μg/ml streptomycin (Thermo Fisher Scientific) for Py230 cells. Cultured cells were harvested using 0.25% trypsin-ethylenediaminetetraacetic acid (EDTA) (Thermo Fisher Scientific), washed through centrifugation (805 g for 5 min) and resuspended in phosphate buffered saline (PBS) (Thermo Fisher Scientific). A Bürcker chamber was used to count the number of harvested cells.

### Intraductal Tumor Cell Inoculation

Lactating female mice were intraductally inoculated in the third mammary gland with either 5 × 10^4^ 4T1 or 1 × 10^5^ Py230 mammary tumor cells under inhalation anesthesia (mixture of 2–3% isoflurane and oxygen). To become lactating, 8-w-old female and male mice were mated and pups were weaned 10 days after delivery. 4T1 and Py230 cells suspended in a mixture of 1:10 PBS and Matrigel® were inoculated through the mammary teat canal 1 h after weaning using a 32-gauge blunt needle in a BALB/c and C57BL/6 background, respectively, to obtain immunocompetent models.

### Analysis of Primary Tumor Growth and Metastases

4T1 and Py230 primary tumor volumes were analyzed weekly as a measurement of primary tumor growth using a digital caliper. The growth of 4T1 primary tumors was also monitored by imaging 4T1-derived bioluminescence at the primary tumor site. Bioluminescence in the Py230 intraductally inoculated NF-κB luciferase reporter mice was monitored at the primary tumor site as a measurement of NF-κB activity and inflammation in primary tumors. To acquire the images, mice were intraperitoneally (i.p.) injected with 200 μl D-luciferin in PBS (at a concentration of 2 mg/100 μl, Gold Biotechnology, St. Louis, MO) and placed in an IVIS lumina II system (PerkinElmer, Zaventem, Belgium) approximately 10 min later under inhalation anesthesia. 4T1-derived bioluminescence in axillary lymph node, lung and liver metastases as well as NF-κB-derived bioluminescence in axillary lymph nodes were acquired through *ex vivo* imaging. Therefore, mice were i.p. injected with a mixture of 100 mg/kg ketamine (Ketamidor, Ecuphar nv/sa, Oostkamp, Belgium) and 10 mg/kg xylazine (Xylazini Hydrochloridum, Val d'Hony-Verdifarm, Beringen, Belgium) for sedation and sacrificed through cervical dislocation. Organs were subsequently isolated and screened for bioluminescence. Spleens were also isolated and weighed. Bioluminescence signals were quantified by dividing the total flux with the selected area using living image analysis software 3.2.

### Flow Cytometric Immunophenotyping of Primary Tumor Immune Populations

4T1 and Py230 primary tumors were isolated from intraductally inoculated mice following scarification. To obtain a single cell suspension, primary tumors were chopped into small fragments and transferred into a gentleMACS C Tube containing an enzyme mix of 2.35 ml RPMI 1640 (Thermo Fisher Scientific), 100 μl Enzyme D, 50 μl Enzyme R and 12.5 μl Enzyme A (enzymes derived from a commercially available Tumor dissociation kit from Miltenyi Biotec, Leiden, The Netherlands). The C Tubes were attached onto a gentleMACS Dissociator, subjected to the gentleMACS Program m_impTumor_02 for 37 s and incubated for 40 min at 37°C with continuous rotation at 40 rpm for optimal enzymatic digestion. The C Tubes were then subjected to the gentleMACS Program m_impTumor_03 twice for 37 s, after which the cell suspensions were allowed to settle and run on the m_impTumor_01 for 60 s to increase the cell yield. Cells were pelleted through a short spin up to 300 g and applied to a 70 μm cell strainer. Washing of the cell strainer with 10 ml of RPMI 1640, pelleting of the cells through centrifugation at 800 g for 7 min and resuspension of the cells into 1 ml of RPMI 1640 created a final cell suspension that was used for further analysis.

Cells were counted on a flow cytometer (Cytoflex, Analis, Ghent, Belgium) by bringing 100 μl of the final cell suspension in a well of a 96 well plate. Cellular viability was also evaluated in this well by adding 2 μl propidium iodide (PI, 50 μg/ml) to the 100 μl suspension. The remaining 900 μl of the final cell suspension was pelleted at 800 g for 7 min and dissolved at a concentration of 2 × 10^6^ cells/ml in FcR blocking reagent diluted 1:10 in FACS buffer (PBS with 1% bovine serum albumin (BSA), 2.5 mM EDTA, and 0.01% sodium azide). For staining of the cells, 100 μl of the mixture (corresponding with 2 × 10^5^ cells) was brought in each well of a 96 well plate, cells were pelleted at 800 g for 5 min and incubated for 30 min at 4°C in a 100 μl cocktail of antibodies supplemented in FACS buffer. Following antibodies and dilutions were used: anti-CD45-biotin (1:200, clone 30-F11, Thermo Fisher Scientific), anti-F4/80-APC (1:20, clone CI:A3-1, Bio-Rad, CA, USA), anti-Ly6G-APC (1:10, clone 1A8, Miltenyi Biotec), anti-CD3ε-FITC (1:50, clone 145-2C11, BioLegend, CA, USA), anti-CD4-PE (1:80, clone GK1.5, BioLegend), anti-CD8a-APC (1:80, clone 53-6.7, BioLegend). Staining with secondary streptavidin-PE (1:333, Thermo Fisher Scientific) or streptavidin-eFluor 450 (1:80, Thermo Fisher Scientific) was performed to detect anti-CD45-biotin. Therefore, cells were pelleted again at 800 g for 5 min, washed with FACS buffer and incubated for 30 min at 4°C in 100 μl of streptavidin antibody supplemented in FACS buffer. Isotype-matched and auto-fluorescence controls were used for identification and quantification of staining positivity. After the cellular stainings, cells were pelleted again at 800 g for 5 min, washed twice with FACS buffer and analyzed with a flow cytometer. All acquired data were processed using CytExpert v2.0.0.153 software (Beckman Coulter, Inc., California, USA). Compensation for spectral overlap between fluorochromes was performed using an automatic calibration technique of the software and subsequently evaluated individually with a matrix.

### Histology and Immunohistochemistry

Tissues were isolated, fixed for 24 h at room temperature (RT) in 3.5% buffered formaldehyde and embedded in paraffin. Tissue sections of 5 μm were deparaffinized, rehydrated and then stained with hematoxylin and eosin (H&E). Following dehydration, tissue sections were mounted with a cover glass for further analysis.

Immunohistochemical stainings relied on antigen retrieval of deparaffinized 2–3 μm thick tissue sections with citrate buffer [pH 6, 10 mM tri-sodium citrate (Santa Cruz Biotechnology, Heidelberg, Germany); for Ki67, CD31, carbonic anhydrase 9 (CAIX), CD45, CD163, CD11c Ly6G, CD3ε, CD8a, CD4, FoxP3, granzyme B, programmed death-1 (PD-1), and CD19], or Tris-EDTA buffer [pH 9, 10 mM Tris, 1 mM EDTA (Thermo Fisher Scientific); for cytokeratin 5] supplemented with 0.05% Tween-20 (Sigma-Aldrich, Bornem, Belgium) at 95°C for 30 min under pressure in a Decloaking Chamber NxGen (Biocare Medical, CA, USA). After cooling down to RT for 30 min, slides were incubated in a microscope slide box with tris-buffered saline (TBS)-wetted paper at 20 rpm on an orbital shaker for further incubation steps. 0.6% H_2_O_2_ in methanol (for CD8a) or 3% H_2_O_2_ in methanol (all other targets) was applied on the sections for 10 min as an endogenous peroxidase block, followed by serum-free protein block for 10 min. Primary antibodies were diluted in Antibody diluent (Dako) and applied for 1 h at RT. Following primary antibodies and dilutions were used: anti-cytokeratin 5 (1:100, clone EP1601Y, Abcam, Cambridge, UK) anti-Ki67 (1:50, clone SP6, Thermo Fisher Scientific), anti-CD31 (1:2000, clone EPR17259, Abcam), anti-CAIX (1:1000, clone NB100-417, Novus Biologicals, Littleton, CO, USA), anti-CD45 (1:1000, clone 30-F11, Thermo Fisher Scientific), anti-CD163 (1:500, clone EPR19518, Abcam), anti-CD11c (1:100, clone D1V9Y, Cell Signaling Technology, Leiden, The Netherlands), anti-Ly6G (1:1000, clone 1A8, BioLegend), anti-CD3ε (1:1000, clone EPR20752, Abcam), anti-CD8a (1:50, clone 4SM15, Thermo Fisher Scientific), anti-CD4 (1:1000, clone 4SM95, Thermo Fisher Scientific), anti-FoxP3 (1:100, clone FJK-16s, Thermo Fisher Scientific), anti-granzyme B (1:1000, polyclonal, Abcam), anti-PD-1 (1:1000, clone EPR20665, Abcam) and anti-CD19 (1:1000, clone 6OMP31, Thermo Fisher Scientific). Secondary antibodies were subsequently applied for 30 min at RT. Following secondary antibodies were used: Rat-on-mouse HRP-Polymer (Biocare Medical) for CD45, Ly6G, CD8a, CD4, FoxP3, and CD19, and Dako EnVision+ Rabbit (Dako) for cytokeratin 5, Ki67, CD31, CAIX, CD163, CD11c, CD3ε, granzyme B, and PD-1. To detect positive staining, sections were treated with a buffer containing 3,3′-diaminobenzidine (DAB) for 10 min at RT. Counterstaining was performed by applying hematoxylin for 5 min. Rinsing of the slides between all incubations relied on TBS applied 3 times for 2 min. For quantification of the stainings, color deconvolution and automatic counting was applied through ImageJ. Ki67 proliferation indices were determined using ImageJS (15).

### Analysis of Cytokine and Protein Levels

To extract proteins, isolated primary tumors and spleens were homogenized and lysed with lysis buffer supplemented with protease inhibitors [1% Nonidet P-40, 10 mM Tris-HCl at pH 7.4, 200 mM NaCl, 5 mM EDTA, 10% glycerol, 100 μM phenylmethylsulfonyl (PMSF), 1 mM oxidized L-glutathione (all from Sigma-Aldrich), 0.15 μM aprotinin and 2.1 μM leupeptin (Roche, Mannheim, Germany)] as previously described (4). Serum was derived from blood which was collected through cardiac puncture, allowed to clot at 37°C for 30 min and centrifuged at 17,000 g for 1 h at 4°C. The levels of 10 selected cytokines (BAFF, G-CSF, IFN-γ, IL-1β, IL-4, IL-6, IL-10, MCP-1, MIP-2, and TNF-α) were measured through Luminex Multiplex Assays (Thermo Fisher Scientific) in primary tumor lysates (50 μg of protein) and sera (1:4 diluted in assay diluent). Enzyme-linked immunosorbent assay (ELISA) was used to measure CHI3L1 and LCN2 levels (Mouse Quantikine ELISA Kit, Biotechne, Minneapolis, MN, USA) in primary tumor lysates, sera and spleen lysates, and the levels of MMP-9, VEGF (Mouse Quantikine ELISA Kit, Biotechne) and TGF-β1 (Mouse uncoated ELISA Kit, Thermo Fisher Scientific) in primary tumor lysates and sera with absorbances at 550 nm subtracted from those at 450 nm for correction. ELISA data were analyzed by means of Deltasoft JV (BioMetallics Incorporated).

### RNA-Seq Analysis

RNA was isolated from 4T1 and Py230 tumors at 3 different time points (i.e., 1, 3, and 6 w p.i.) using in house developed protocols (16). All analyses were done in triplicate. In addition, Matrigel® controls were included for each intraductal model and time point, yielding a total of 36 samples. RNA quality and quantity were analyzed using the Agilent BioAnalyzer and the NanoDrop, respectively, according the manufacturer's instructions. RNA sequencing was done in collaboration with Oxford Genomics using Illumina's TruSeq chemistry. Briefly, 100 ng of total RNA was subjected to a stranded library preparation, and libraries were sequenced (i.e., paired-end; read length of 75 bp) on a HiSeq4000 using 2 lanes aiming for 10 million reads per sample. This allows accurate assessment of the expression levels of the 50% most strongly expressed genes (coefficient of variation between 10 replicates inferior to 5%), which corresponds to genes having at least 10 associated reads in the respective libraries. Reads were mapped to the mouse reference genome (mm10) using STAR. Read counts were generated using the summariseOverlaps function from the BioConductor package GenomicAlignments. Raw counts were normalized and analyzed for differential expression using the DESeq2 package in BioConductor. Regression models were set up to allow evaluation of expression differences between the 4T1- and Py230-based intraductal model at each time point and plain time effects were accounted for using the Matrigel® control samples. Significant differences were considered when nominal *P*-values were inferior to 5%. Lists of differentially expressed genes were translated into biological concepts (i.e., hallmark category of the molecular signatures database from the Broad Institute) using gene set enrichment analysis (GSEA). Therefore, mouse gene symbols were first translated into their human homologs using the getLDS function from the biomaRt package in BioConductor. Heatmaps in **Figures 10**, **11** were generated using the online tool Heatmapper (17) and in Supplementary Figure 5 using R.

### Statistical Analysis

Analysis of Variance (ANOVA) tests with Tukey's *post-hoc* tests and unpaired Student's *t*-tests were performed using Prism (GraphPad) to calculate *P*-values and determine statistical significant differences (*P* < 0.05). Log_10_ normalization was performed to normalize data when necessary.

## Results

### DCIS to IC Progression Is More Aggressive in the 4T1- Compared to the Py230-Based Intraductal Model as Reflected by Local and Systemic Disease Markers

**Figure 1:**
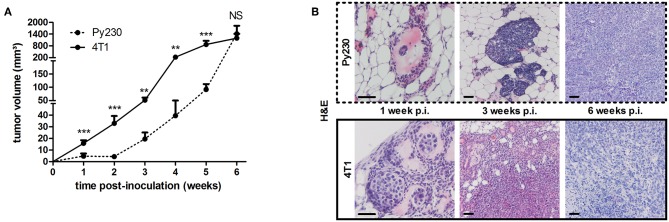
Tumor growth, ductal breakthrough and tumor proliferation in the 4T1- compared to Py230-based intraductal model. 4T1 and Py230 mammary tumor cells were intraductally inoculated in syngeneic lactating dams and primary tumor progression was monitored. **(A)** Weekly primary tumor volume measurements up to 6 w p.i. (4T1 primary tumors: *n* = 52 at 1 w p.i., *n* = 36 at 2 and 3 w p.i., *n* = 18 at 4, 5, and 6 w p.i.; Py230 primary tumors: *n* = 43 at 1 w p.i., *n* = 33 at 2 and 3 w p.i., *n* = 23 at 4 and 5 w p.i., *n* = 29 at 6 w p.i.). **(B)** H&E histology of Py230 and 4T1 primary tumors at 1, 3, and 6 w p.i. Scale bars = 50 μm. Data in **(A)** are presented as the means ± standard error of the mean (SEM). NS, not significant. **P < 0.01, ***P < 0.001.

**Figure 2:**
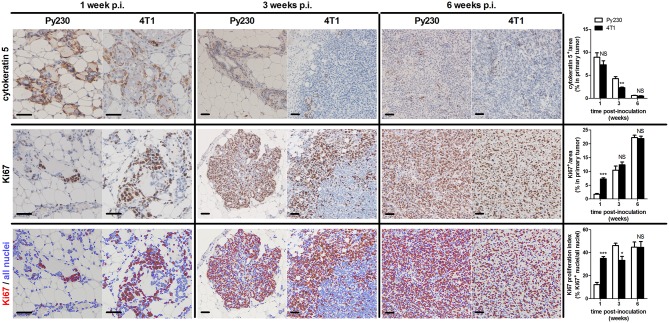
Ductal breakthrough and tumor proliferation in the 4T1- compared to Py230-based intraductal model. Immunohistochemistry for the myoepithelial cell marker cytokeratin 5 and cell proliferation marker Ki67 on sections of Py230 and 4T1 primary tumors at 1, 3, and 6 w p.i. (*n* = 5 at each time point for both 4T1 and Py230 primary tumors). A Ki67 proliferation index was determined of Py230 and 4T1 primary tumors at 1, 3, and 6 w p.i. by calculating the fraction of Ki67^+^ nuclei among all nuclei using ImageJS (15). All nuclei are marked in blue and Ki67^+^ nuclei are marked in red. Scale bars = 50 μm. Data are presented as the means ± SEM. NS, not significant. *P < 0.05, **P < 0.01, ***P < 0.001.

To establish the immunocompetent 4T1- and Py230-based intraductal model, BALB/c-derived 4T1 mammary tumor cells and C57BL/6-derived Py230 mammary tumor cells were intraductally inoculated in lactating syngeneic mice. Primary tumor volume measurements indicated that 4T1 cells grew more aggressively than Py230 cells inside the mammary glands and established larger primary tumors between 1 and 5 w p.i. (Figure 1A). In contrast, Py230 cells grew exponentially and at 6 w p.i. the Py230 and 4T1 primary tumor volumes no longer differed (Figure 1A). As the 4T1 tumor cells were luminescently traceable, *in vivo* imaging allowed to verify their invasive primary tumor growth and showed that they expanded across the mammary gland and the surrounding tissue (Supplementary Figure 1A). H&E histology of primary tumors at 1, 3, and 6 w p.i. confirmed DCIS to IC progression in both the 4T1- and Py230-based intraductal model, with 4T1 cells displaying both enhanced and earlier invasion at 3 w p.i. (Figure 1B). Corroborating this key finding, increased disruption of the myoepithelial cell layer by 4T1 cells at 3 w p.i. was verified based on decreased cytokeratin 5 positivity in 4T1 compared to Py230 primary tumors (Figure 2). Ki67 staining confirmed the increased tumor cell proliferation in 4T1 compared to Py230 primary tumors at 1 w p.i., but not at 3 and 6 w p.i. (Figure 2). Moreover, calculation of the Ki67 proliferation index (i.e., the percentage of Ki67 stained nuclei relative to all nuclei in the primary tumor) showed that, in marked contrast to the index at 1 w p.i., Py230 primary tumors were more proliferative than 4T1 primary tumors by 3 w p.i. (Figure 2), supporting the earlier finding that Py230 tumors are able to catch up with 4T1 tumor growth.

**Figure 3:**
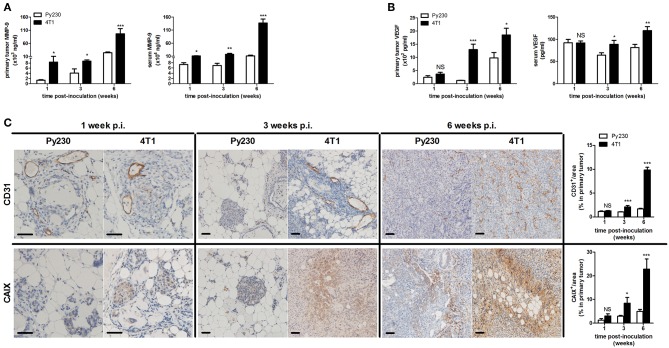
MMP-9 and VEGF levels, tumor vascularity and hypoxia in the 4T1- compared to Py230-based intraductal model. Primary tumor and serum MMP-9 **(A)** and VEGF **(B)** levels at 1, 3, and 6 w p.i. in the 4T1- and Py230-based intraductal model (4T1-based model: *n* = 7 tumors and 10 sera at 1 w p.i., *n* = 7 tumors and 9 sera at 3 w p.i., *n* = 8 tumors and 9 sera at 6 w p.i.; Py230-based model: *n* = 7 tumors and 10 sera at 1 w p.i., *n* = 5 tumors and 7 sera at 3 w p.i., *n* = 11 tumors and 7 sera at 6 w p.i.). **(C)** Immunohistochemistry for the endothelial cell marker CD31 and hypoxia marker CAIX on sections of 4T1 and Py230 primary tumors at 1, 3, and 6 w p.i. (*n* = 5 at each time point for both 4T1 and Py230 primary tumors). Scale bars = 50 μm. Data are presented as the means ± SEM. NS, not significant. *P < 0.05, **P < 0.01, ***P < 0.001.

**Figure 4:**
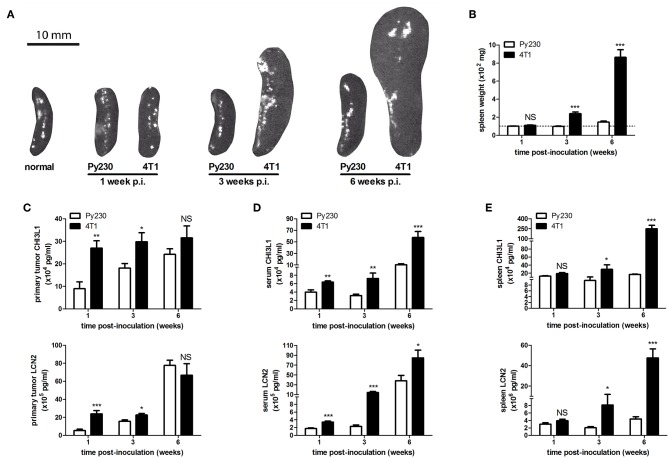
Splenomegaly and levels of immune-related disease biomarkers CHI3L1 and LCN2 in the 4T1- compared to Py230-based intraductal model. **(A)** Representative images of spleens isolated from the 4T1- and Py230-based intraductal model at 1, 3, and 6 w p.i. The image of a spleen from a normal mouse is also shown for comparison. **(B)** Weight measurements of spleens isolated from the 4T1- and Py230-based intraductal model at 1, 3, and 6 w p.i. (*n* = 9 spleens at each time point for both the 4T1- and Py230-based model). The dotted line indicates the mean spleen weight of 6 normal mice. **(C–E)** CHI3L1 and LCN2 levels at 1, 3, and 6 w p.i. in primary tumors **(C)**, sera **(D)**, and spleens **(E)** of the 4T1- and Py230-based intraductal model (4T1-based model: *n* = 6 tumors, 8 sera, and 5 spleens at 1 w p.i., *n* = 7 tumors, 8 sera, and 5 spleens at 3 w p.i., *n* = 8 tumors, 8 sera, and 6 spleens at 6 w p.i.; Py230-based model: *n* = 7 tumors, 9 sera, and 5 spleens at 1 w p.i., *n* = 5 tumors, 7 sera, and 5 spleens at 3 w p.i., *n* = 11 tumors, 7 sera, and 5 spleens at 6 w p.i.). Data in **(B–E)** are presented as the means ± SEM. NS, not significant. *P < 0.05, **P < 0.01, ***P < 0.001.

Additionally, distant metastases in the 4T1- and Py230-based intraductal model were investigated at 6 w p.i. by H&E histology and *ex vivo* imaging. H&E histology showed metastases in axillary lymph nodes, lungs and liver from 4T1, but not from Py230 tumor-bearing mice (Supplementary Figure 1B). *Ex vivo* imaging confirmed the presence of 4T1-derived bioluminescence in these three organs (Supplementary Figure 1B). MMP-9 and VEGF, proteins that are important for tumor metastasis through extracellular matrix degradation and angiogenesis, respectively, were measured locally in primary tumors and systemically in serum, identifying increased MMP-9 levels at 1, 3, and 6 w p.i. (Figure 3A) and increased VEGF levels at 3 as well as 6 w p.i. (Figure 3B) in the 4T1- compared to the Py230-based intraductal model. However, MMP-9 and VEGF levels in primary tumors and serum did not increase to the same extent. The fold induction of the mean MMP-9 and VEGF levels in the 4T1- compared to the Py230-based model showed remarkable differences in primary tumors (Supplementary Figures 2A,B). More specifically, MMP-9 was more strongly induced at 1 w p.i. compared to VEGF, whereas at 3 w p.i. VEGF was more strongly induced compared to MMP-9 (Supplementary Figures 2A,B). At 6 w p.i., MMP-9 was again more strongly induced compared to VEGF, albeit to a lesser extent than at 1 w p.i. (Supplementary Figures 2A,B). In serum, the fold induction of MMP-9 in the 4T1- compared to the Py230-based model was only clearly higher compared to that of VEGF at 6 w p.i. (Supplementary Figures 2A,C). CD31 stainings in primary tumors corroborated the increased angiogenesis at 3 and 6 w p.i. in the 4T1-based intraductal model (Figure 3C). However, based on stainings for the hypoxia marker CAIX (18, 19) the increased angiogenesis did not result in a decrease of hypoxia. More specifically, 4T1 primary tumors showed a progressive increase in CAIX positivity from 1 to 6 w p.i., whereas the slower progressing Py230 primary tumors showed fewer CAIX staining (Figure 3C). The increased tumor progression in the 4T1-based intraductal model was accompanied with a more progressive splenomegaly compared to the Py230-based intraductal model based on spleen weight measurements at 1, 3, and 6 w p.i. (Figures 4A,B), indicative for enhanced systemic disease as well as leukemoid reactions (20, 21). Two immuno-oncological biomarkers for disease monitoring in mice (3, 4) and breast cancer patients (22–24), CHI3L1 and LCN2, corroborated the 4T1 disease progression and showed significantly increased levels in 4T1 compared to Py230 primary tumors at 1 and 3 w p.i., but not at 6 w p.i. (Figure 4C), when 4T1 and Py230 primary tumors reached similar volumes. In serum, on the other hand, CHI3L1 and LCN2 were significantly increased at 1, 3, and 6 w p.i. in the 4T1- compared to the Py230-based intraductal model (Figure 4D), reflecting the enhanced metastatic progression of 4T1 tumor cells following intraductal inoculation. CHI3L1 and LCN2 levels in spleens corroborated the increased splenomegaly at 3 and 6 w p.i. in the 4T1- compared to the Py230-based intraductal model (Figure 4E) and further indicated the presence of enhanced leukocyte reactions in the 4T1 tumor-bearing mice.

### DCIS to IC Progression Is Accompanied by an Increase in Immune Cells That Occurs Earlier in the 4T1- Compared to the Py230-Based Intraductal Model

**Figure 5:**
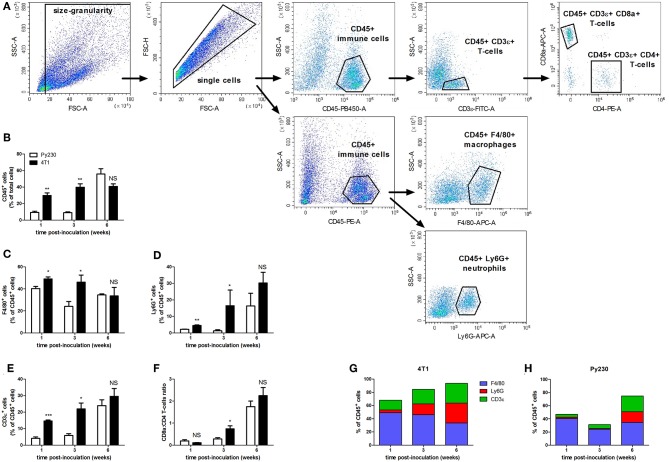
Flow cytometric immunophenotyping of tumor immune populations in the 4T1- compared to Py230-based intraductal model. Primary tumors were isolated from the 4T1- and Py230-based intraductal model at 1, 3, and 6 w p.i. and dissociated into a single cell suspension. Distinct immune cell types in the single cell suspension were quantified by flow cytometry. **(A)** Gating strategy for analysis of single cells and expression of CD45, F4/80, Ly6G, CD3ε, CD8a, and CD4. **(B–F)** Positivity for CD45 (pan-immune cells) as a percentage of total single cells **(B)**, positivity for F4/80 (macrophages) **(C)**, Ly6G (neutrophils) **(D)**, and CD3ε (T-cells) **(E)** as a percentage of CD45^+^ cells, and the ratio of CD8a^+^ cells (cytotoxic T-cells) and CD4^+^ cells (T-helper cells) **(F)** at 1, 3, and 6 w p.i. in primary tumors isolated from the 4T1- and Py230-based intraductal model (4T1 primary tumors: *n* = 4 at each time point; Py230 primary tumors: *n* = 4 at 1 w p.i., *n* = 3 at 3 and 6 w p.i.). F4/80^+^, Ly6G^+^, and CD3ε^+^ fraction of total leukocytes in Py230 **(G)** and 4T1 **(H)** primary tumors at 1, 3, and 6 w p.i. Data in **(B–F)** are presented as the means ± SEM, data in **(G,H)** are presented as the means. NS, not significant. *P < 0.05, **P < 0.01, ***P < 0.001.

The immune system plays a pivotal role in the progression of mammary tumors. Moreover, their invasiveness is associated with immune cell changes in the tumor microenvironment (25, 26). In order to investigate the primary tumor immune cell populations upon DCIS to IC progression in the 4T1- and Py230-based intraductal model, isolated primary tumors from both these models at 1, 3 and 6 w p.i. were processed into single cells and immunophenotyped by flow cytometry (Figure 5A). Based on the pan-immune cell marker CD45, the number of leukocytes increased over time in the 4T1 as well as the Py230 primary tumors (Figure 5B). However, at 1 and 3 w p.i. a significantly higher number of CD45^+^ immune cells was detected in 4T1 compared to Py230 primary tumors, indicative for a substantially more enriched tumor immune microenvironment in the 4T1-based intraductal model at these early time points (Figure 5B). In accordance with the higher percentage of CD45^+^ immune cells, 4T1 primary tumors showed significantly increased percentages of the F4/80^+^ (Figure 5C), Ly6G^+^ (Figure 5D), and CD3ε^+^ (Figure 5E) immune cell types at 1 and 3 w p.i. compared to Py230 primary tumors. When the CD3ε^+^ T-cell population was subdivided into CD4^+^ T-helper cells and CD8a^+^ cytotoxic T-cells, an increase in the CD8a/CD4 T-cell ratio was found in 4T1 as well as Py230 primary tumors between 1 and 6 w p.i. (Figures 5A,F). Consequently, DCIS to IC progression is associated with a switch from a high number of T-helper cells to a high number of cytotoxic T-cells in the primary tumor microenvironment. However, the significantly increased CD8a/CD4 T-cell ratio at 3 w p.i. in 4T1 compared to Py230 primary tumors indicates that this switch occurs earlier in the 4T1-based intraductal model. Investigation of the changes in proportion of immune cells over time in the 4T1- and Py230-based intraductal model showed that F4/80^+^ macrophages were the most abundant immune cell type at 1 and 3 w p.i., and that Ly6G^+^ neutrophils and CD3ε^+^ T-cells caught up with the macrophage numbers by 6 w p.i. (Figures 5G,H). More specifically, in 4T1 primary tumors, each of these three immune cell types comprised about 30% of the total immune cells at this end point, together approximating 90% of the primary tumor immunophenotype (Figure 5G). In marked contrast, in the Py230-based primary tumors, F4/80^+^ macrophages remained the predominant immune cell type (34%) at 6 w p.i., although closely followed in numbers by Ly6G^+^ neutrophils (16%) and CD3ε^+^ T-cells (24%), together approximating 75% of the primary tumor immunophenotype (Figure 5H).

**Figure 6:**
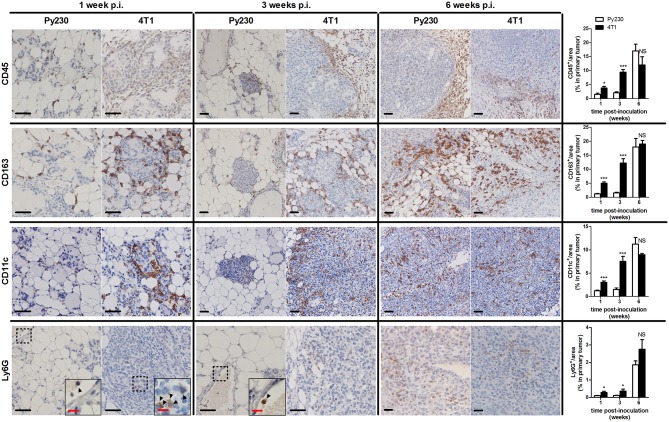
Immunohistochemical analysis of innate immune cell populations in primary tumors of the 4T1- compared to Py230-based intraductal model. Immunohistochemistry for the pan-immune cell marker CD45, the tumor-associated macrophage marker CD163, the dendritic cell marker CD11c and the neutrophil marker Ly6G on sections of 4T1 and Py230 primary tumors at 1, 3, and 6 w p.i. (*n* = 5 at each time point for both 4T1 and Py230 primary tumors). Dashed inserts and arrowheads indicate the few Ly6G^+^ cells at 1 w p.i. in both 4T1 and Py230 primary tumors and at 3 w p.i. in Py230 primary tumors. Black scale bar = 50 μm, red scale bar = 20 μm. Data are presented as the means ± SEM. NS, not significant. *P < 0.05, ***P < 0.001.

**Figure 7:**
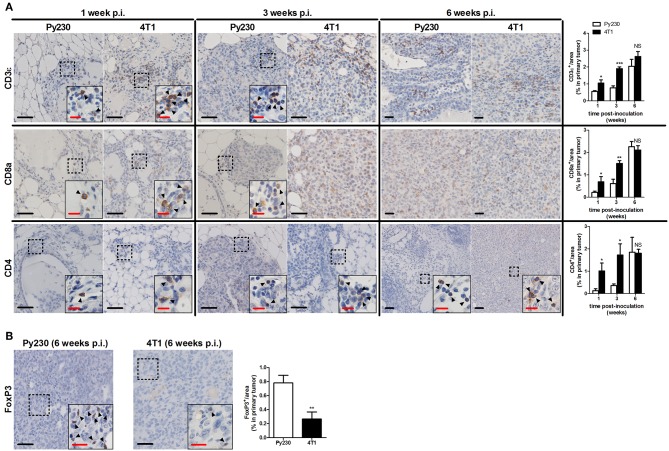
Immunohistochemical analysis of adaptive immune cell populations in primary tumors of the 4T1- compared to Py230-based intraductal model. **(A)** Immunohistochemistry for the T-cell marker CD3ε, the cytotoxic T-cell marker CD8a and the T-helper cell marker CD4 on sections of 4T1 and Py230 primary tumors at 1, 3, and 6 w p.i. (*n* = 5 at each time point for both 4T1 and Py230 primary tumors). **(B)** Immunohistochemistry for the regulatory T-cell marker FoxP3 on sections of 4T1 and Py230 primary tumors at 6 w p.i. (*n* = 5 at each time point for both 4T1 and Py230 primary tumors). Dashed inserts and arrowheads indicate the few CD3ε^+^ and CD8a^+^ cells at 1 w p.i. in both 4T1 and Py230 primary tumors and at 3 w p.i. in Py230 primary tumors, the few CD4^+^ cells at 1, 3, and 6 w p.i. in both 4T1 and Py230 primary tumors, and the few FoxP3^+^ cells at 6 w p.i. in 4T1 and Py230 primary tumors. Black scale bar = 50 μm, red scale bar = 20 μm. Data are presented as the means ± SEM. NS, not significant. *P < 0.05, **P < 0.01, ***P < 0.001.

Immunohistochemical CD45 stainings corroborated the immunophenotyping by identifying an increased number of immune cells over time in the tumor stroma, but also immune cells infiltrating into the tumor tissue at 3 and 6 w p.i. (Figure 6), when tumor cells became invasive. The 4T1 primary tumors showed significantly increased CD45 positivity at 1 and 3 w p.i. and equal CD45 positivity at 6 w p.i. compared to Py230 primary tumors. Similarly, CD163 stainings demonstrated a progressive increase of tumor-associated macrophages (TAMs) in the surrounding primary tumor stroma, with 4T1 primary tumors showing significantly higher CD163 positivity at 1 and 3 w p.i., but not at 6 w p.i. compared to Py230 primary tumors (Figure 6). Also CD11c and Ly6G stainings for tumor-associated dendritic cells (TADCs) and tumor-associated neutrophils (TANs), respectively, increased over time in primary tumor sections of both models, with 4T1 primary tumors showing significantly increased positivity for both markers at 1 and 3 w p.i., but not at 6 w p.i. compared to Py230 primary tumors (Figure 6). Moreover, whereas Ly6G stainings were located within the primary tumor area at all times, CD11c stainings for TADCs were initially located in the tumor stroma at 1 w p.i. and subsequently invaded the primary tumor core by 3 and 6 w p.i. Regarding the adaptive immune cells, immunohistochemistry showed significant increase in CD3ε stainings for tumor-infiltrating lymphocytes (TILs), in CD8a stainings for cytotoxic T-cells, and in CD4 stainings for T-helper cells in the primary tumor area of the 4T1- compared to Py230-based model at 1 and 3 w p.i., but not at 6 w p.i. (Figure 7A). FoxP3 stainings for immunosuppressive regulatory T-cells significantly increased in the Py230 compared to 4T1 primary tumors at 6 w p.i. (Figure 7B).

### More Aggressive Tumor Progression in the 4T1- Compared to the Py230-Based Intraductal Model Is Characterized by an Enhanced Inflammation and Cytotoxic T-Cell Activity

Mammary tumor progression is highly influenced by inflammatory responses derived from activated immune cells in the tumor microenvironment. This inflammation is tightly controlled by inflammatory transcription factors such as NF-κB (27). NF-κB luciferase reporter mice allow to measure the host NF-κB activation upon tumor progression and visualize mammary gland inflammation through bioluminescence imaging (11, 28, 29). Py230 cells were intraductally inoculated in these reporter mice and weekly monitoring identified that host NF-κB activity at the primary tumor site exponentially increased over time, although there was a distinctive signal only at 6 w p.i. (Supplementary Figures 3A,B). Isolated axillary lymph nodes from the mice at 6 w p.i. also showed significantly higher NF-κB-derived luminescence compared to 1 and 3 w p.i. (Supplementary Figures 3C,D), indicative for an increasing inflammation in response to the advancing primary tumor.

**Figure 8:**
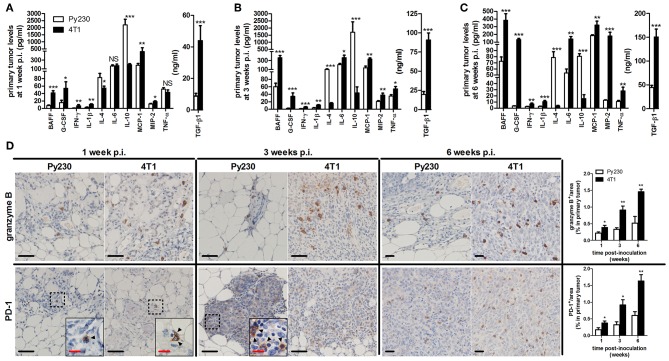
Cytokine levels and immunohistochemical analysis of cytotoxic T-cell activity in primary tumors of the 4T1- compared to Py230-based intraductal model. Cytokine levels at 1 w p.i. **(A)**, 3 w p.i. **(B)**, and 6 w p.i. **(C)** in primary tumors of the 4T1- and Py230-based intraductal model (4T1 primary tumors: *n* = 7 at 1 and 3 w p.i., *n* = 8 at 6 w p.i.; Py230 primary tumors: *n* = 7 at 1 w p.i., *n* = 5 at 3 w p.i., *n* = 11 at 6 w p.i.). **(D)** Immunohistochemistry for the activated cytotoxic T-cell markers granzyme B and PD-1 (*n* = 5 at each time point for both 4T1 and Py230 primary tumors). Dashed inserts and arrowheads indicate the few PD-1^+^ cells at 1 w p.i. in both 4T1 and Py230 primary tumors and at 3 w p.i. in Py230 primary tumors. Black scale bar = 50 μm, red scale bar = 20 μm. Data are presented as the means ± SEM. NS, not significant. *P < 0.05, **P < 0.01, ***P < 0.001.

Local and systemic cytokine profiling allowed a more in-depth investigation into the inflammation in the intraductal model. 4T1 primary tumors showed significantly increased levels of pro-inflammatory cytokines B-cell activating factor (BAFF), granulocyte colony-stimulating factor (G-CSF), interferon (IFN)-γ, interleukin (IL)-1β, monocyte chemoattractant protein (MCP)-1, macrophage inflammatory protein (MIP)-2, and transforming growth factor (TGF)-β1 at 1, 3, and 6 w p.i., and IL-6 and tumor necrosis factor (TNF)-α at 3 and 6 w p.i. compared to Py230 primary tumors (Figures 8A–C). In contrast, IL-4 and IL-10 levels were significantly higher in Py230 compared to 4T1 primary tumors at all 3 time points (Figures 8A–C). Analysis of cytokine levels in serum provided similar results as in primary tumors with significantly higher BAFF, G-CSF, MCP-1, MIP-2, and TGF-β1 levels at 1, 3, and 6 w p.i. for the 4T1- compared to the Py230-based intraductal model (Supplementary Figures 4A–C). IFN-γ, IL-6, and TNF-α serum levels were detectable only at 3 and 6 w p.i. and also significantly increased in the 4T1- compared to the Py230-based intraductal model (Supplementary Figures 4A–C).

Cytokine production and release is linked to immune cell activation in the tumor microenvironment, which can be targeted with immunotherapy. Especially cytotoxic (CD8a^+^) T-cells are an important immunotherapeutic target due to their tumor-killing function. Therefore, comparative evaluation of the cytotoxic T-cell activity in primary tumors of both intradutcal models is of major added value before these models can be used as therapeutic screening tools. Immunohistochemical stainings for granzyme B and PD-1, both markers for activated cytotoxic T-cells, showed increased positivity in 4T1 compared to Py230 primary tumors at 1, 3, and 6 w p.i. (Figure 8D). The latter key observation is in accordance with the higher pro-inflammatory cytokine levels in the 4T1- compared to the Py230-based intraductal model. CD19 stainings further revealed the presence and increase of activated B-cells at 3 and 6 w p.i. in 4T1, but not in Py230 primary tumors (Supplementary Figure 4D).

### RNA-Seq Verifies Immunological and Proliferative Changes Upon Tumor Progression in the 4T1- Compared to the Py230-Based Intraductal Model

**Figure 9:**
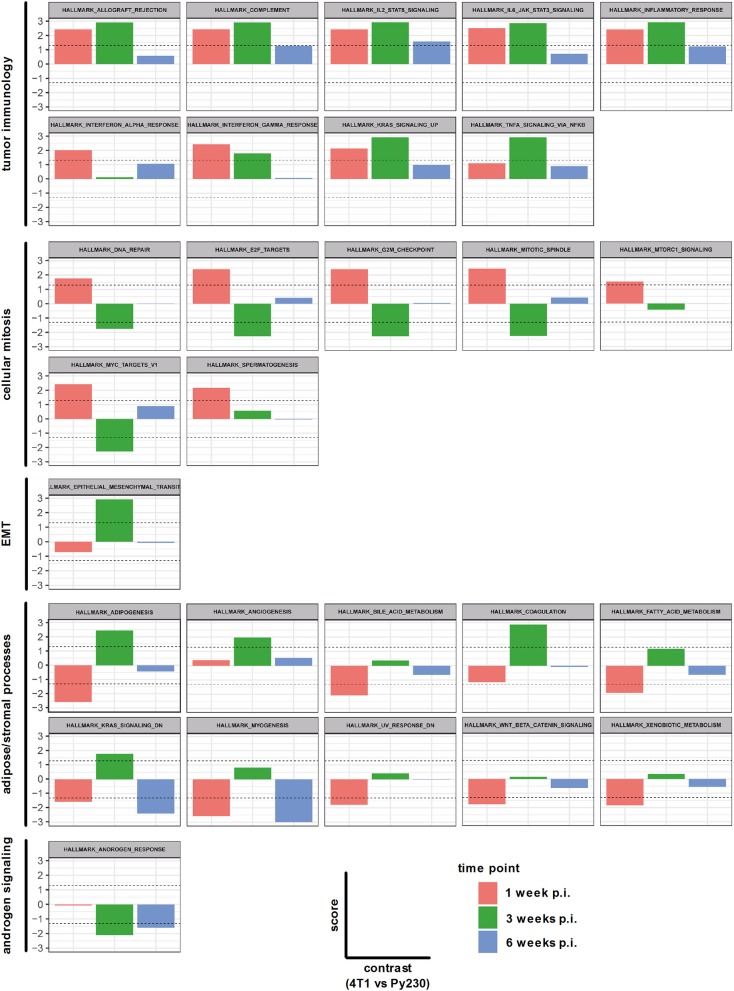
Hallmarks associated with differentially expressed genes in primary tumors of the 4T1- compared to Py230-based intraductal model. RNA isolated from 4T1 and Py230 primary tumors at 1, 3, and 6 w p.i. was sequenced and differentially expressed genes were categorized into hallmark gene sets using GSEA. The hallmark gene sets were related to tumor immunology, cellular mitosis, EMT, adipose/stromal processes, and androgen signaling. The x-axis in each graph subdivides the gene set expression in 4T1 compared to Py230 primary tumors at 1, 3, and 6 w p.i. The y-axis in each graph indicates the score (corresponding with the *P*-value in log_10_ scale) for the gene set expression in 4T1 compared to Py230 primary tumors. More specifically, a positive score identifies that the gene set is higher expressed in 4T1 than in Py230 primary tumors, whereas a negative score identifies the opposite. The dashed lines indicate the minimal level of significance (= 0.05 or 1.3 in log_10_ scale) and allow to identify whether the difference in expression of the hallmark gene set between 4T1 and Py230 primary tumors is statistically significant.

In order to investigate the changes in both intraductal models upon tumor progression at the genetic level, sequencing was performed on RNA isolated from snap frozen 4T1 and Py230 intraductally inoculated primary tumors at 1, 3 and 6 w p.i. The differentially expressed genes in the 4T1 vs. Py230 primary tumor datasets at the 3 different time points were categorized into 28 hallmark gene sets using GSEA. Nine hallmarks could be related to tumor immunology and collectively showed a decreased expression over time (allograft rejection, complement, IL2-STAT5 signaling, IL-6-JAK-STAT3 signaling, inflammatory response, IFN-α response, IFN-γ response, KRAS signaling up, and TNF-α signaling via NF-κB) (Figure 9). More specifically, all 9 tumor immunology-related gene sets remained upregulated in 4T1 compared to Py230 primary tumors across the 3 time points, but only significantly at 1 and/or 3 w p.i. Other gene sets could be related to cellular mitosis and tumor progression, including DNA repair, E2F targets, G2M checkpoint, mitotic spindle, MTORC1 signaling, MYC targets V1 and spermatogenesis (Figure 9). Whereas at 1 w p.i. all gene sets were significantly upregulated, at 3 w p.i. they became significantly downregulated or showed a strong decrease compared to 1 w p.i. in 4T1 compared to Py230 primary tumors, indicative for decreased tumor proliferation. At 6 w p.i., the gene sets went back to baseline, highlighting the similar tumor size in 4T1 and Py230 primary tumors. In contrast to the cellular mitosis gene sets, the hallmark epithelial-mesenchymal transition (EMT) was significantly upregulated at 3 w p.i. in 4T1 compared to Py230 primary tumors (Figure 9), indicative for metastatic progression. Gene sets involved in adipose/stromal processes within the mammary gland (adipogenesis, angiogenesis, bile acid metabolism, coagulation, fatty acid metabolism, KRAS signaling dn, myogenesis, UV response dn, WNT-β-catenin signaling, and xenobiotic metabolism) were downregulated or weakly expressed at 1 w p.i., but became upregulated at 3 w p.i. in 4T1 compared to Py230 primary tumors (Figure 9), indicating that at the time 4T1 tumor cells were less proliferative compared to Py230 tumor cells, the surrounding stroma and fat tissue were more active in the 4T1- compared to the Py230-based intraductal model. The hallmark androgen response also showed a significantly upregulated expression in Py230 tumors at 3 and 6 w p.i. (Figure 9), indicating that androgen signaling is important in invasive Py230 tumors. Hierarchical clustering of the hallmarks in a heatmap with the different primary tumor samples at the different time points confirmed that tumor immunology hallmarks are most represented in 4T1 primary tumors, with the highest representation at 1 w p.i., and cellular mitosis hallmarks are most represented in 4T1 primary tumors at 6 w p.i. and in Py230 primary tumors at 3 and 6 w p.i. (Supplementary Figure 5). Gene expression of RNA samples derived from Matrigel®-only intraductally injected lactating BALB/c and C57BL/6 mice at 1, 3, and 6 w p.i. were also included as a control and showed a strong representation of stromal/adipose-related hallmarks (Supplementary Figure 5), indicative for an active involution process following lactation. The control samples did not show upregulation of immunological hallmarks, which suggests that the injection method and Matrigel® have minimal influence on tumor immunology.

**Figure 10:**
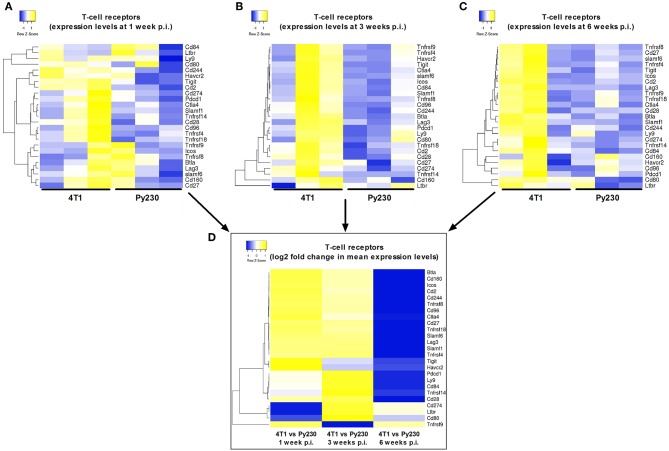
Differential expression of T-cell receptors in primary tumors of the 4T1- compared to Py230-based intraductal model. Heatmap displaying expression levels of selected genes involved in co-stimulatory/co-inhibitory T-cell receptors in three independent 4T1 and Py230 primary tumor samples at 1 w p.i. **(A)**, 3 w p.i. **(B)**, and 6 w p.i. **(C)**. **(D)** Heatmap displaying the log2 fold change in mean gene expression of the selected co-stimulatory/co-inhibitory T-cell receptors in 4T1 and Py230 primary tumor samples at 1, 3, and 6 w p.i. The selection of the T-cell receptor genes was based on the gene list from the NanoString Mouse PanCancer Immune Profiling Panel and a publication by Yu et al. (30). Hierarchical clustering was performed using Pearson distance.

**Figure 11:**
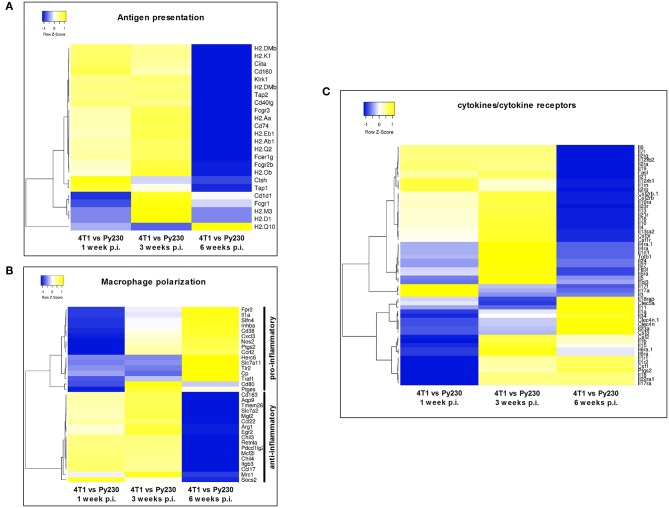
Differential expression of antigen presentation, macrophage polarization and cytokines/cytokine receptor gene sets in primary tumors of the 4T1- compared to Py230-based intraductal model. Heatmaps displaying the log2 fold change in mean gene expression of selected genes involved in antigen presentation, macrophage polarization and cytokines/cytokine receptors in 4T1 and Py230 primary tumor samples at 1, 3, and 6 w p.i. The selection of the genes was based on the gene list from the NanoString Mouse PanCancer Immune Profiling Panel and a publication by Yu et al. (30). Genes that characterize pro-inflammatory and anti-inflammatory macrophage polarization were selected based on a publication by Jablonski et al. (31). Hierarchical clustering was performed using Pearson distance.

Next, a specific gene set for co-stimulatory and co-inhibitory T-cell receptors was investigated at 1, 3, and 6 w p.i. in 4T1 and Py230 primary tumors. Individual heatmaps were generated for the expression levels at each time point and every heatmap indicated increased T-cell receptor expression in most, but not all 4T1 compared to Py230 primary tumors (Figures 10A–C). Yet, the difference in T-cell receptor gene expression between the 4T1 and Py230 primary tumors decreased over time (Figure 10D). Furthermore, gene sets for antigen presentation, macrophage polarization and cytokines/cytokine receptors were also investigated. Based on the heatmap, differential expression of genes involved in antigen presentation strongly decreased at 6 w p.i. in 4T1 compared to Py230 primary tumors (Figure 11A). Gene expression data also showed that 4T1 primary tumors change from an anti-inflammatory at 1 w p.i. to a pro-inflammatory macrophage gene signature at 6 w p.i. compared to Py230 primary tumors (Figure 11B). This finding was further supported by upregulation of pro- and downregulation of anti-inflammatory cytokines and their receptors at 6 w p.i. in 4T1 compared to Py230 primary tumors (Figure 11C).

## Discussion

Inoculation of murine mammary tumor cells in the teat duct is increasingly accepted as an alternative to the classical fat pad inoculation for studying breast cancer. The added value of such intraductally established tumors is that they grow from within the mammary ducts and first undergo ductal breakthrough prior to their invasion of the mammary fat pad and their metastasis to other organs, which more closely resembles the disease process in humans (3–5). The use of fully immunocompetent syngeneic mice further allows to study the influence of the immune system, which has recently been recognized to play a key role in mammary tumor progression (25, 26). In this context, we have previously shown that macrophages are critical regulators of the DCIS to IC progression and stimulate metastasis in the 4T1-based syngeneic intraductal model for TNBC (4). With the current comparative study, we aimed to gain deeper insight in the immunological changes underlying aggressive 4T1 vs. non-aggressive Py230 intraductal tumor progression, providing further characterization of the intraductal model prior to its envisaged use for evaluation of novel breast cancer therapeutics.

Following intraductal inoculation, 4T1 tumor cells initially grew faster and showed earlier ductal breakthrough than Py230 tumor cells, reproducibly metastasizing to axillary lymph nodes, lungs and livers at 6 w p.i., in marked contrast to the absence of metastases in the Py230-based intraductal model. Although it was not checked how long it takes for the Py230-based intraductal model to induce detectable metastases, previous studies reported that it may take up to 20 weeks for the development of Py230 lung metastases (14). Indeed, the Py230 tumor cell line is not susceptible to EMT, being an important feature to gain migratory and hence metastatic capacity (32, 33).

At 6 w p.i., 4T1 and Py230 primary tumors had a similar size, which could be explained by their marked difference in Ki67 positivity and Ki67 proliferation index, reflecting a decreased proliferation of 4T1 in time compared to Py230 primary tumors. Indeed, 4T1 tumors are typically only highly proliferative at the edges being a hallmark of their aggressive invasive and metastatic character (34) while their core remains poorly proliferative due to a lack of essential nutrients and oxygen (30). Our data further demonstrated that Py230 tumors were able to catch up with 4T1 tumor growth by proliferating both at the tumor core and the edges. Complementary RNA-seq analysis could attribute this decreased proliferation of 4T1 compared to Py230 primary tumors to the significant downregulation of genes involved in cellular mitosis at 3 w p.i. At that time point, a significant upregulation of genes involved in EMT and adipogenic/stromal processes indicated that 4T1 cells focus on both EMT and stromal development for metastasis rather than on primary tumor growth compared to Py230 cells. The increased expression of genes related to adipogenesis and stromal processes can also be explained by the fact that both 4T1 and Py230 cells were injected in actively involuting mammary glands. Lactation makes the teat orifice accessible for intraductal inoculation without surgery or microscopic guidance (3, 5). Moreover, it offers a representative model for pregnancy-associated breast cancer, which is associated with high mortality rates in young women and has remained understudied till date (35, 36).

The aggressive metastatic progression of the 4T1-based tumors was further associated with increased local and systemic levels of MMP-9, an essential matrix degrading protein that paves the way for invasion, and of VEGF, which is an important driver of blood vessel development for sustaining oxygen and nutrient supply and for transporting tumor cells to distant organs. Of relevance, the kinetic variation of MMP-9 and VEGF levels in their local fold inductions showed a remarkable resemblance with the differential primary tumor growth between the TNBC models and the RNA-seq data. Indeed, at 1 w p.i., when 4T1 primary tumors were significantly more proliferative than Py230 primary tumors and cellular mitosis hallmarks were significantly increased in 4T1 compared to Py230 primary tumors, the MMP-9 levels were also more induced, allowing the fast expansion and ductal breakthrough of 4T1 tumor cells. However, at 3 w p.i., 4T1 primary tumors had become more oriented toward adipose/stromal processes, including angiogenesis, resulting in a significant induction of VEGF. In line with these results, the hallmark angiogenesis was most strongly and significantly upregulated at 3 w p.i. in 4T1 compared to Py230 primary tumors, as shown in the RNA-seq data. At 6 w p.i., local MMP-9 was again more induced compared to VEGF, which may correspond with enhanced matrix degradation to allow metastatic progression. However, this late MMP-9 induction was lower than the early one at 1 w p.i., which may be due to the similar tumor sizes and cellular mitosis at 6 w p.i. in 4T1- vs. Py230-based primary tumors. In marked contrast, the VEGF induction at 6 w p.i remained similar to that at 1 w p.i., corroborating the 4T1- vs. the Py230-based primary tumor RNA-seq data for the hallmark angiogenesis. The increased fold induction of MMP-9 compared to VEGF at 6 w p.i. in serum was in line with the increased systemic disease at that endpoint in the 4T1- compared to the Py230-based TNBC model as also shown by the enhanced metastasis and splenomegaly data.

Similar to VEGF levels, CD31 positivity identified enhanced vascularity in the 4T1-based intraductal model. CAIX positivity reflected the increased hypoxia in 4T1 primary tumors corroborating the decreased proliferation in the 4T1 tumor core, which the 4T1 tumor cells try to alleviate by stimulating angiogenesis for enhanced oxygen and nutrient supply, and by increasing CAIX production for pH regulation (19). An alternative hypoxia biomarker is pimonidazole, which needs to be injected 1–2 h before scarification of the tumor-bearing mice and has been regarded as more superior because of this exogenous use in contrast to the endogenously produced CAIX (37, 38). Yet, several studies have reported a good agreement and only minor variations between both these hypoxia stainings on tumor sections from different cancer types, including breast cancer (39). Upon side-by-side comparison of CAIX and pimonidazole stainings on 4T1 tumor sections in our group, we also observed similar patterns and tumor hypoxia (data not shown). In marked contrast to 4T1 primary tumors, Py230 tumors showed only limited CAIX and CD31 positivity, indicative for a more oxygen-enriched and nutritious tumor environment enhancing cell viability and proliferation both at the tumor core and edges, stimulating a fast and controlled tumor outgrowth. In line with our results, Yang et al. also categorized 4T1 tumors as highly angiogenic and invasive, but weakly proliferative compared to other mammary tumor cell lines following orthotopic immunocompetent inoculation in the mammary fat pad (40).

Progressing 4T1 tumors are associated with severe splenomegaly due to granulocytic hyperplasia (20, 21). Our results also showed how spleen sizes significantly increased over time in the 4T1-based intraductal model, whereas in the Py230-based intraductal model spleens remained almost at a normal size, suggesting differential leukemoid reactions in both models. CHI3L1 and LCN2, two immune-related biomarkers used for monitoring intraductal tumor progression (3, 4) and breast cancer patient prognosis (22–24), confirmed the differential tumor progression and immunology with differential levels in spleens, primary tumors and serum of both models. Immunophenotyping identified an increase of immune cells in the 4T1 and Py230 primary tumors that corresponded to the ductal mammary tumor progression in each intraductal model, corroborating previous studies that associated immune cell infiltration with tumor cell invasion in breast cancer (41, 42). Both the abundance, localization and type of immune cell populations were further investigated through immunohistochemistry. Most immune cells surrounded the mammary tumors, including CD163^+^ TAMs. These TAMs are one of the most abundant type of immune cells in 4T1 and Py230 primary tumors at 1 w p.i., corroborating previous studies in mice and humans (30, 42) and highlighting their involvement in DCIS to IC progression as shown recently by our group (4). Others have also shown that macrophage depletion reduces tumor growth and invasion at early stages (43–46). Over time, more Ly6G^+^ TANs and CD3ε^+^ TILs were found within the primary tumor mass. The role of these cell types in DCIS to IC transition is not well-known, yet both pro- and anti-tumor properties have been attributed to them (47–49). CD11c^+^ TADCs were also increasingly found over time within the tumor mass in both intraductal models and are important in T-cell activation through their antigen presenting function. As CD11c^+^ TADCs and TILs are strongly correlated in TNBC (50), the increase in TADCs corroborates with an upregulation of CD3ε^+^, CD8a^+^, and CD4^+^ cells in primary tumors. Moreover, as both 4T1 and Py230 tumors were infiltrated with TILs, and more specifically immunoreactive CD8a^+^ cytotoxic T-cells and CD4^+^ T-helper cells, they can be classified as so called “hot” tumors (51). Yet, FoxP3^+^ regulatory T-cells were upregulated in Py230 compared to 4T1 primary tumors, highlighting a strongly immunosuppressed and proliferative microenvironment in the Py230-based model. The progressive NF-κB activation in Py230 primary tumors and draining axillary lymph nodes corroborated the increases in tumor immune cell populations over time.

Cytokine profiles further corroborated the enhanced local and systemic leukemoid responses in the 4T1- compared to Py230-based intraductal model. More specifically, 4T1 primary tumors showed a stimulated pro-inflammatory cytokine profile including IL-1β, IL-6, IFN-γ, and TNF-α, suggesting a cytotoxic immune microenvironment with T-cell activation. Although increased TGF-β1 levels in the 4T1-based intraductal model might indicate immunosuppression, they can also be attributed to 4T1 tumor invasion (52). In marked contrast, Py230 primary tumors showed a stimulated anti-inflammatory cytokine profile including increased IL-10 and IL-4 levels at all time points, corroborating the enhanced regulatory T-cell positivity and immunosuppressive properties of Py230 primary tumors. Moreover, based on granzyme B and PD-1 positivity, 4T1 tumors showed significantly stronger activation of cytotoxic T-cells compared to Py230 tumors, in line with the differential cytokine profiles.

An important remaining question is what regulates and determines the immunological differences between both intraductal TNBC models. An important tumor cell extrinsic factor that should not be neglected and could have influenced the tumor immunology is the mouse strain-dependent immune bias. Indeed, BALB/c mice are notoriously Th2 skewed and produce potent antibody responses, which is one of the reasons that this is the strain of choice for hybridoma production, allergy studies and anti-parasite studies (30, 53–55). On the other hand, C57BL/6 mice are much more Th1 prone, which is the reason why this strain is frequently used for viral and tumor studies (30, 53–55). Yet, the current study shows that C57BL/6-derived Py230 primary tumors are more immunosuppressive compared to BALB/c-derived 4T1 primary tumors, demonstrating that tumor cell intrinsic or other extrinsic factors overruled the strain-specific immunity in regulating the mammary tumor immune responses and phenotype. Of note, other frequently used C57BL/6-derived tumor cell lines are known to be highly inflammatory compared to BALB/c-derived tumors (40, 56), demonstrating that not every tumor in C57BL/6 mice is less inflammatory and again suggesting that intrinsic/extrinsic factors play a key role in shaping tumor immunity in mouse models. The specific environment in which tumor cells grow can also highly impact on the tumor immunology and immunotherapeutic responses (30). To this end, orthotopic implantation of tumor cells has been reported to establish a highly immunosuppressive tumor phenotype that is less responsive to immunotherapy compared to subcutaneously implanted tumors (30, 57). Since the mammary ducts are considered as the truly orthotopic injection site for mammary tumor cells (5, 8), the intraductal model is most likely to resemble the correct immunophenotype associated with implanted mammary tumor cell lines and also mimic tumor immunology and therapeutic responses observed in patients. Furthermore, it may be a concern that only 4T1 cells carried a luciferase reporter gene, which as a foreign gene could influence the differential tumor immunology between the 4T1- and Py230-based intraductal model. However, previous reports have shown that luciferase is a very weak antigen and does not significantly alter tumor progression (58, 59). Moreover, 4T1 cells are also intrinsically weakly immunogenic and have a limited mutational burden (40, 58).

Further corroborating the cytokine profiles and the reports that T-cell activation may decrease tumor proliferation, RNA-seq also identified increased tumor-associated immune responses in 4T1 compared to Py230 primary tumors. However, there was a decreasing trend over time in all immunology-related gene sets, indicating that Py230 primary tumors were catching up with 4T1 tumor immunology. This finding was also highlighted by gene sets for T-cell receptors and antigen presentation, showing a decrease over time in differential gene expression between 4T1 and Py230 primary tumors. Interestingly, CD274, also referred to as the immune checkpoint protein programmed death-ligand 1 (PD-L1), was one of the few genes that remained upregulated at 6 w p.i. compared to 1 w p.i., suggesting that the PD-L1 axis plays a critical role in 4T1 tumor progression. The latter increase in expression also corroborates the increased protein levels of IFN-γ, an important inducer for PD-L1 (60). Nevertheless, Mosely et al. reported that anti-PD-L1 treatment of 4T1 tumors fails to induce an effective therapeutic response (56). Upon further investigation these authors identified that granulocytic myeloid-derived suppressor cells (MDSCs) were present in high numbers in these 4T1 primary tumors, highlighting excessive granulocytosis in the 4T1-based intraductal model (56). The decreased expression of anti-inflammatory and increased expression of pro-inflammatory cytokines and macrophage markers over time in 4T1 compared to Py230 primary tumors again confirmed that Py230 tumors are highly immunosuppressed and likely will be even more refractory to checkpoint inhibitors. It can be suggested that in order to overcome immunosuppression and allow effective immunotherapy in both intraductal models for TNBC, an additional layer of treatment may be required, such as chemotherapy, radiotherapy, agonists of T-cell co-stimulatory receptors or vaccines (56, 61, 62). Alternatively, Li et al. recently reported that TNBC can be eradicated by targeting glycosylated PD-L1, inducing both immune re-activation as well as 4T1 tumor cell killing (63). The current study also showed a remarkable upregulation of genes related to androgen signaling in Py230 primary tumors, indicating that androgens play an important role in intraductal Py230 tumor outgrowth (64). It can therefore be suggested that inhibition of androgen receptor signaling may provide a beneficial therapeutic effect in the Py230-based intraductal model for TNBC. Of relevance and in line with this hypothesis, a subset of TNBC patients shows high expression of the androgen receptor and remains difficult to treat, but shows good responses to anti-androgen therapy (65).

Taken together, the 4T1- and Py230-based intraductal model are characterized by a different tumor outgrowth and associated tumor microenvironment. More specifically, whereas 4T1 tumors are aggressively metastatic and show high levels of EMT, hypoxia and inflammation, Py230 tumors remain locally invasive and show high proliferation and immunosuppression. These differential models may broadly represent the clinically observed TNBC diversity and together provide a powerful tool to evaluate immunotherapy and therapeutic combinations in a truly orthotopic environment at both early (DCIS) or late stage (IC) breast carcinoma.

## Data Availability Statement

The RNA-seq data has been uploaded to GEO (Gene-Expression Omnibus)—the accession number is GSE140192. Other raw data supporting the conclusions of this manuscript will be made available by the authors, without undue reservation, to any qualified researcher.

## Ethics Statement

The animal study was reviewed and approved by the Committee on the Ethics of Animal Experiments of The Faculty of Veterinary Medicine at Ghent University (approval numbers: EC2015/127, EC2016/56 and EC2017/80).

## Author Contributions

JS, SV, and EM concepted and designed the study. JS, NV, and KD acquired the data. JS, NV, KD, and SV analyzed and interpreted the data. JS drafted the manuscript. OD, NS, WV, LD, SV, and EM critically revised the manuscript. JS, NV, KD, OD, NS, WV, LD, SV, and EM gave their approval of the final manuscript.

### Conflict of Interest

The authors declare that the research was conducted in the absence of any commercial or financial relationships that could be construed as a potential conflict of interest.

## Supplementary Material

Supplementary Figure 1 *In vivo* imaging of 4T1 primary tumor progression and identification of metastases in the 4T1- and Py230-based intraductal model. **(A)** Representative image of the *in vivo* bioluminescence as a measurement of primary tumor growth in 4T1 intraductally inoculated mice at 1, 3, and 6 w p.i. **(B)** H&E histology for the identification of metastases in axillary lymph nodes, lungs, and liver from 4T1 and Py230 intraductally inoculated mice at 6 w p.i. Dashed inserts show a magnification of tumor tissue in the 4T1-based intraductal model and normal tissue in the Py230-based intraductal model. White dashed lines in the axillary lymph node image derived from the 4T1-based intraductal model indicate the border between normal and tumor tissue. Black scale bar = 100 μm; red scale bar = 50 μm. Representative images of *ex vivo* bioluminescence derived from 4T1 metastases at 6 w p.i. in axillary lymph nodes, lungs, and liver are also shown.

Supplementary Figure 2 Fold induction of MMP-9 and VEGF levels in the 4T1- compared to Py230-based intraductal model. **(A)** Table showing the mean MMP-9 and VEGF levels measured in primary tumors and serum of the 4T1- and Py230-based intraductal model at 1, 3, and 6 w p.i. as displayed in Figures 3A,B. The relative fold induction of mean MMP-9 and VEGF levels at each time point in 4T1- compared to Py230-derived primary tumors and serum was calculated and displayed in red. **(B,C)** Graphs displaying the calculated fold induction of mean MMP-9 and VEGF levels at 1, 3, and 6 w p.i. in primary tumors **(B)** and serum **(C)** of the 4T1- compared to the Py230-based intraductal model.

Supplementary Figure 3 Host NF-κB activity upon progression of intraductally inoculated Py230 tumor cells in NF-κB reporter mice. **(A)** Representative image of the *in vivo* bioluminescence as a measurement of host NF-κB activation in Py230 intraductally inoculated NF-κB reporter mice at 1, 3, and 6 w p.i. **(B)** Host NF-κB activation in Py230 primary tumors up to 6 w p.i. based on weekly measurements of the total flux density at the inoculation sites (*n* = 20 tumors at 1 w p.i., *n* = 10 tumors at all other time points). **(C)** Representative image of the *ex vivo* bioluminescence in axillary lymph nodes of Py230 intraductally inoculated NF-κB reporter mice at 1, 3, and 6 w p.i. **(D)** Host NF-κB activation in axillary lymph nodes of Py230 intraductally inoculated NF-κB reporter mice at 1, 3, and 6 w p.i. based on measurements of the total flux density (*n* = 4 axillary lymph nodes at 1 and 3 w p.i., *n* = 5 axillary lymph nodes at 6 w p.i.). Data are presented as the means ± SEM. ^*^*P* < 0.05, ^**^*P* < 0.01, ^***^*P* < 0.001.

Supplementary Figure 4 Serum cytokine levels in the 4T1- compared to Py230-based intraductal model and immunohistochemical analysis of activated B-cells in 4T1 primary tumors. Cytokine levels at 1 w p.i. **(A)**, 3 w p.i. **(B)**, and 6 w p.i. **(C)** in serum of the 4T1- and Py230-based intraductal model (BAFF, G-CSF, IFN-γ, IL-6, MCP-1, MIP-2, TNF-α: *n* = 5 at each time point and for both 4T1 and Py230 sera; TGF-β1: *n* = 8 for 4T1 and *n* = 5 for Py230 at each time point). **(D)** Immunohistochemistry for the activated B-cell marker CD19 on sections of 4T1 and Py230 primary tumors at 1, 3, and 6 w p.i. (*n* = 5 at each time point for both 4T1 and Py230 primary tumors). Scale bar = 50 μm. Data are presented as the means ± SEM. NS, not significant. ^*^*P* < 0.05, ^**^*P* < 0.01, ^***^*P* < 0.001.

Supplementary Figure 5 Heatmap related to the hallmarks identified in Figure 9. The heatmap displays the expression of the hallmarks from Figure 9 in 4T1 and Py230 primary tumors and Matrigel®-only inoculated mammary glands at 1, 3, and 6 w p.i. (*n* = 3 at each time point for 4T1 and Py230 primary tumors and Matrigel®-only inoculated BALB/c- and C57BL/6-derived mammary glands). Hierarchical clustering was performed using Euclidean distance.
