# Supplementary figures and images for: Comparative Profiling of Metastatic 4T1- vs. Non-metastatic Py230-Based Mammary Tumors in an Intraductal Model for Triple-Negative Breast Cancer
Source: Front Immunol. 2019 Dec 17;10:2928. doi: 10.3389/fimmu.2019.02928 (PMC6927949; doi:10.3389/fimmu.2019.02928)

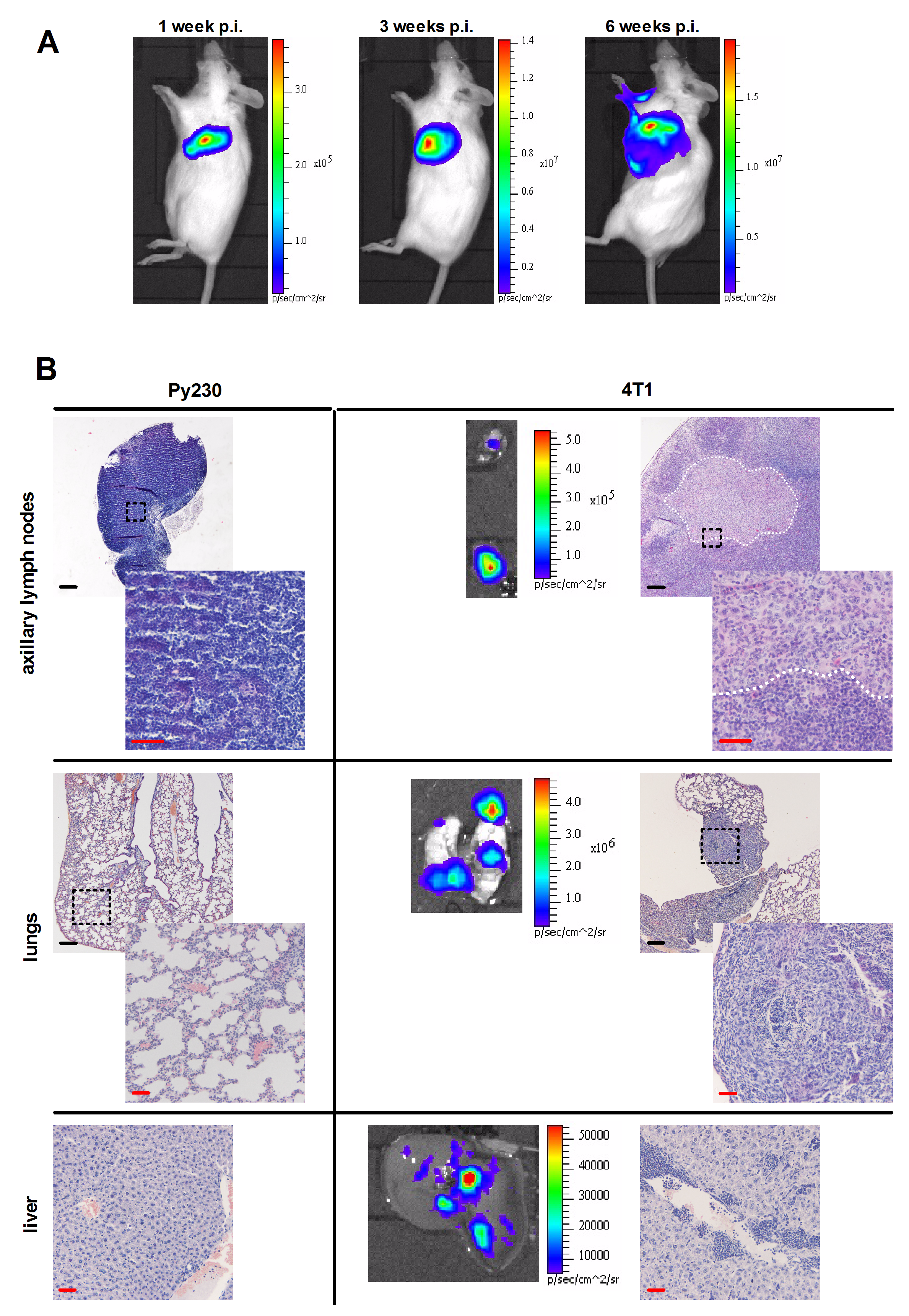

Supplement: Supplementary Figure 1 — In vivo imaging of 4T1 primary tumor progression and identification of metastases in the 4T1- and Py230-based intraductal model. (A) Representative image of the in vivo bioluminescence as a measurement of primary tumor growth in 4T1 intraductally inoculated mice at 1, 3, and 6 w p.i. (B) H&E histology for the identification of metastases in axillary lymph nodes, lungs, and liver from 4T1 and Py230 intraductally inoculated mice at 6 w p.i. Dashed inserts show a magnification of tumor tissue in the 4T1-based intraductal model and normal tissue in the Py230-based intraductal model. White dashed lines in the axillary lymph node image derived from the 4T1-based intraductal model indicate the border between normal and tumor tissue. Black scale bar = 100 μm; red scale bar = 50 μm. Representative images of ex vivo bioluminescence derived from 4T1 metastases at 6 w p.i. in axillary lymph nodes, lungs, and liver are also shown. [file Image_1.TIF]

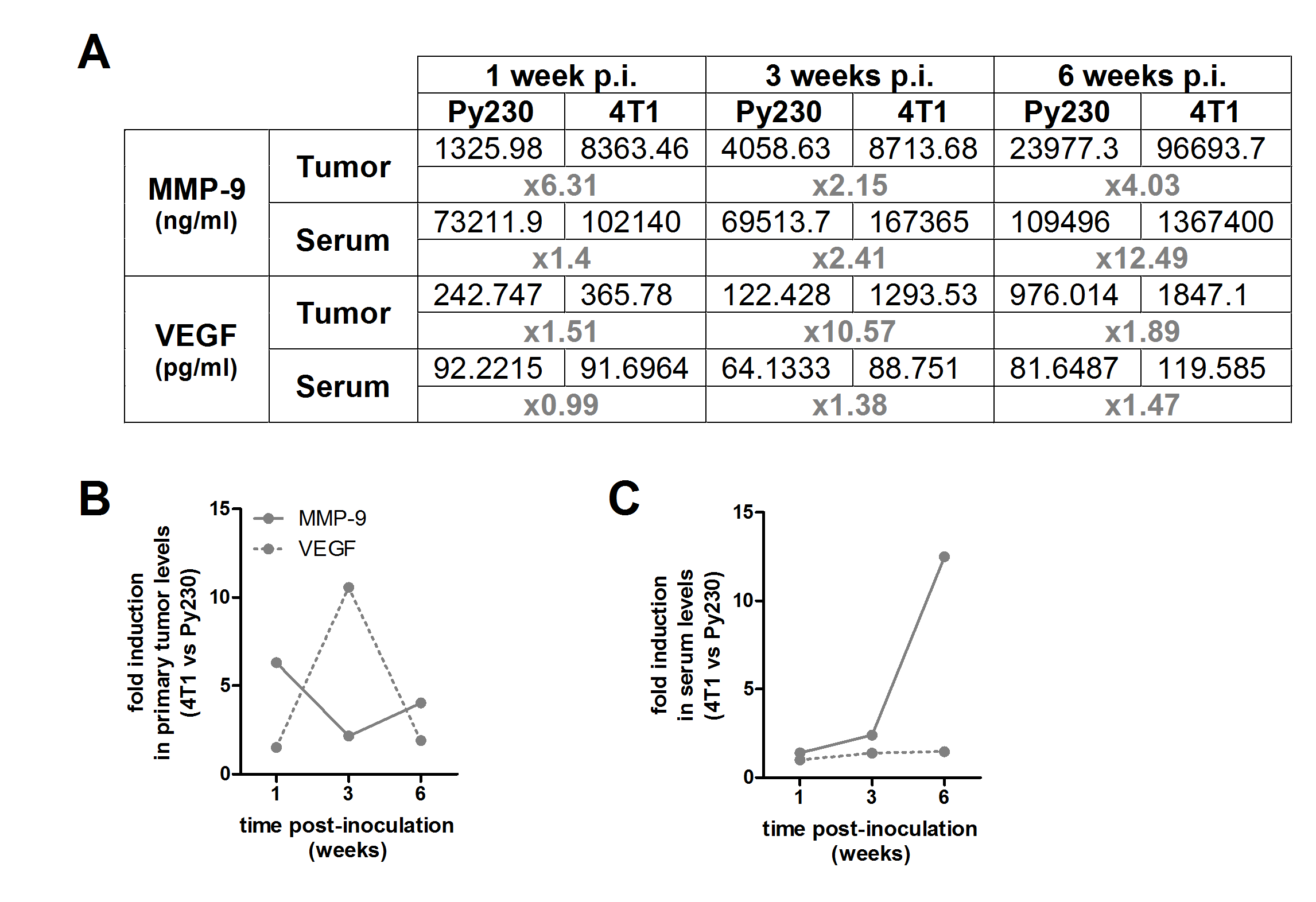

Supplement: Supplementary Figure 2 — Fold induction of MMP-9 and VEGF levels in the 4T1- compared to Py230-based intraductal model. (A) Table showing the mean MMP-9 and VEGF levels measured in primary tumors and serum of the 4T1- and Py230-based intraductal model at 1, 3, and 6 w p.i. as displayed in Figures 3A,B. The relative fold induction of mean MMP-9 and VEGF levels at each time point in 4T1- compared to Py230-derived primary tumors and serum was calculated and displayed in red. (B,C) Graphs displaying the calculated fold induction of mean MMP-9 and VEGF levels at 1, 3, and 6 w p.i. in primary tumors (B) and serum (C) of the 4T1- compared to the Py230-based intraductal model. [file Image_2.TIF]

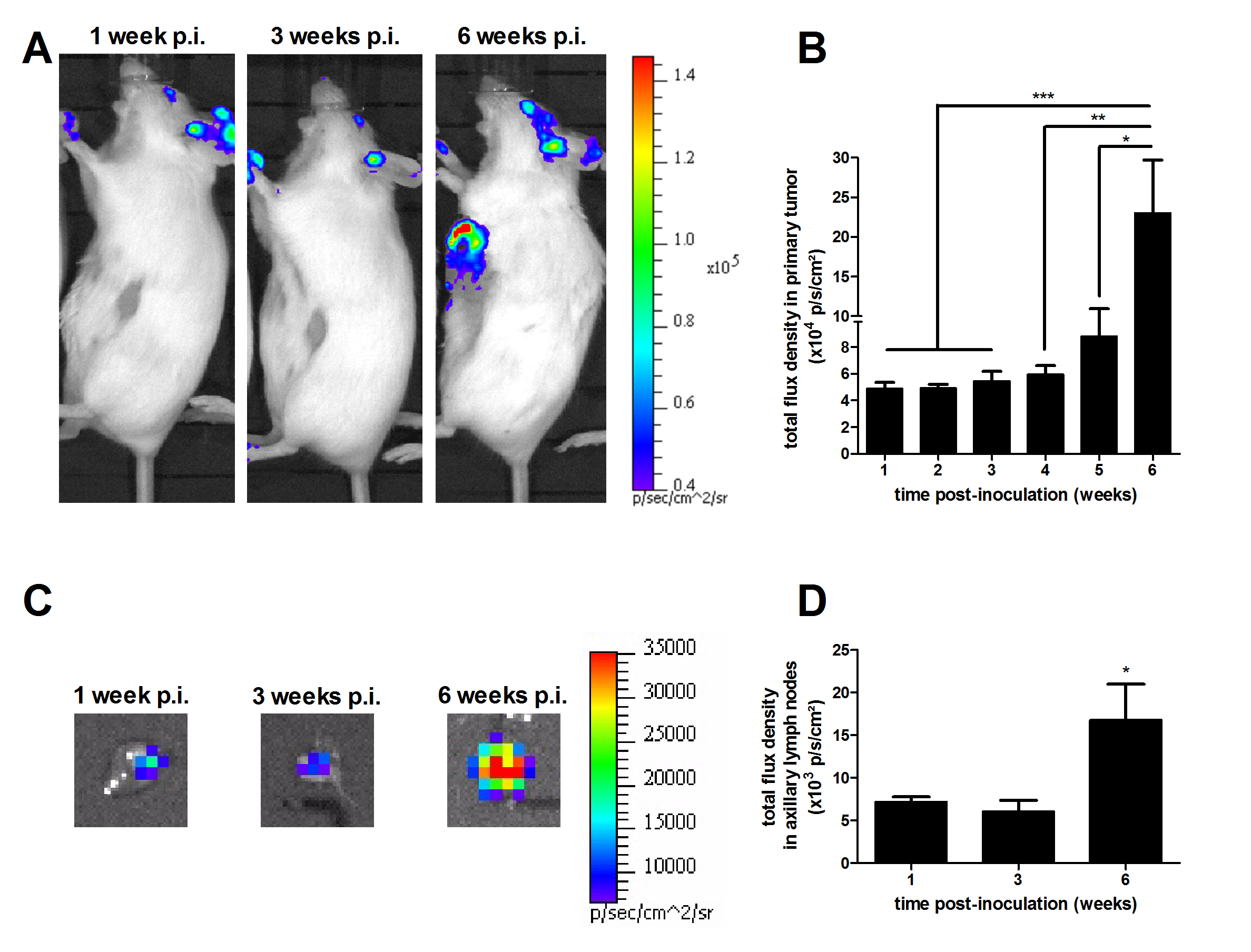

Supplement: Supplementary Figure 3 — Host NF-κB activity upon progression of intraductally inoculated Py230 tumor cells in NF-κB reporter mice. (A) Representative image of the in vivo bioluminescence as a measurement of host NF-κB activation in Py230 intraductally inoculated NF-κB reporter mice at 1, 3, and 6 w p.i. (B) Host NF-κB activation in Py230 primary tumors up to 6 w p.i. based on weekly measurements of the total flux density at the inoculation sites (n = 20 tumors at 1 w p.i., n = 10 tumors at all other time points). (C) Representative image of the ex vivo bioluminescence in axillary lymph nodes of Py230 intraductally inoculated NF-κB reporter mice at 1, 3, and 6 w p.i. (D) Host NF-κB activation in axillary lymph nodes of Py230 intraductally inoculated NF-κB reporter mice at 1, 3, and 6 w p.i. based on measurements of the total flux density (n = 4 axillary lymph nodes at 1 and 3 w p.i., n = 5 axillary lymph nodes at 6 w p.i.). Data are presented as the means ± SEM. *P < 0.05, **P < 0.01, ***P < 0.001. [file Image_3.TIF]

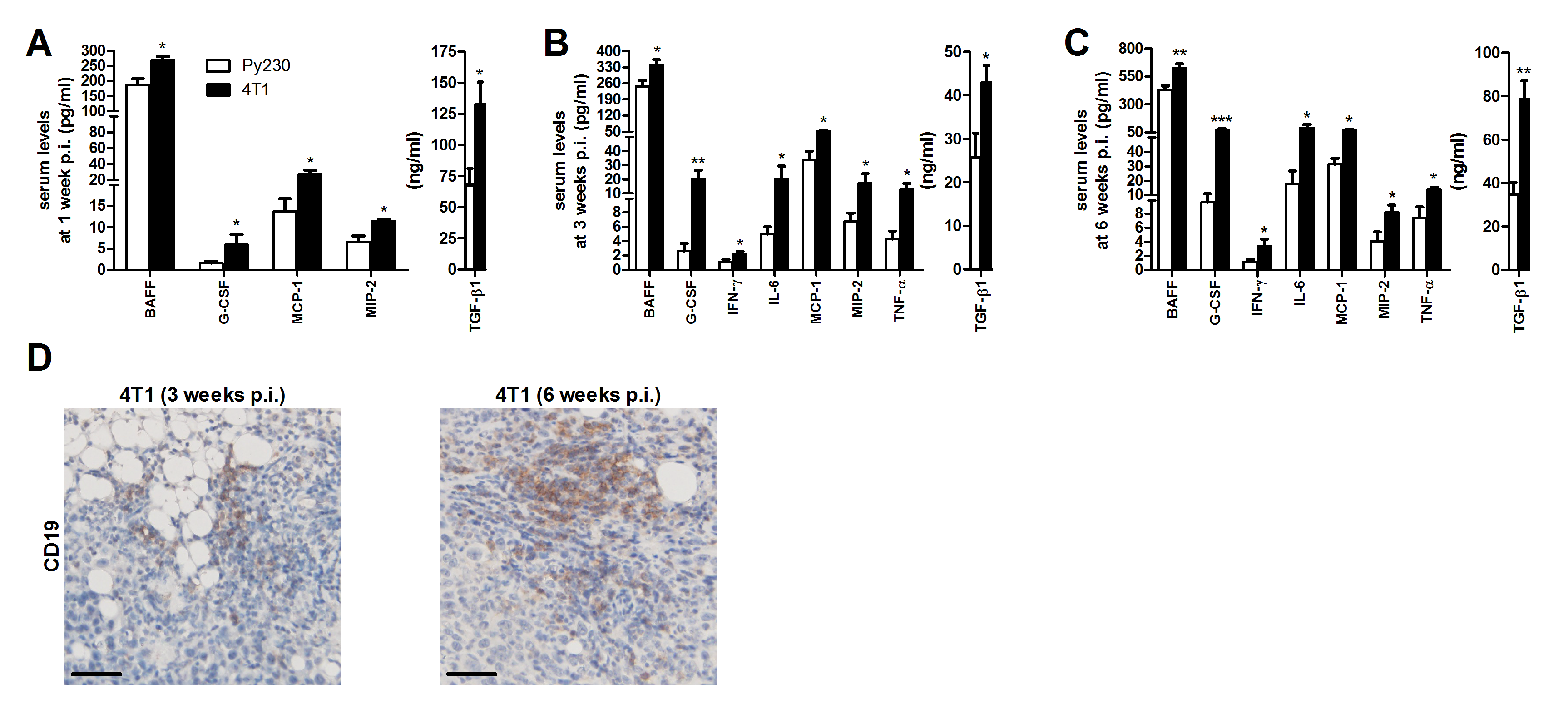

Supplement: Supplementary Figure 4 — Serum cytokine levels in the 4T1- compared to Py230-based intraductal model and immunohistochemical analysis of activated B-cells in 4T1 primary tumors. Cytokine levels at 1 w p.i. (A), 3 w p.i. (B), and 6 w p.i. (C) in serum of the 4T1- and Py230-based intraductal model (BAFF, G-CSF, IFN-γ, IL-6, MCP-1, MIP-2, TNF-α: n = 5 at each time point and for both 4T1 and Py230 sera; TGF-β1: n = 8 for 4T1 and n = 5 for Py230 at each time point). (D) Immunohistochemistry for the activated B-cell marker CD19 on sections of 4T1 and Py230 primary tumors at 1, 3, and 6 w p.i. (n = 5 at each time point for both 4T1 and Py230 primary tumors). Scale bar = 50 μm. Data are presented as the means ± SEM. NS, not significant. *P < 0.05, **P < 0.01, ***P < 0.001. [file Image_4.TIF]

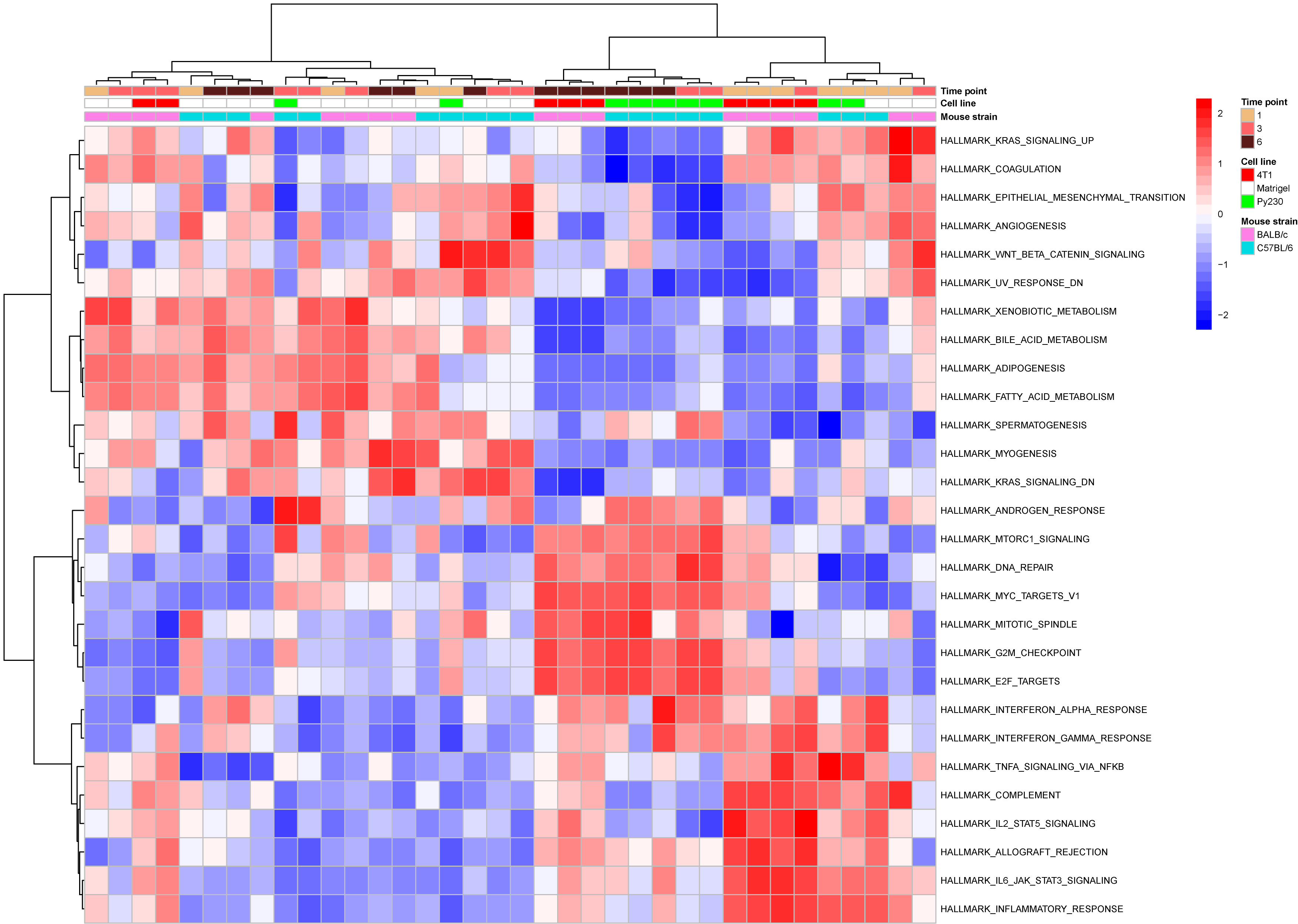

Supplement: Supplementary Figure 5 — Heatmap related to the hallmarks identified in Figure 9. The heatmap displays the expression of the hallmarks from Figure 9 in 4T1 and Py230 primary tumors and Matrigel®-only inoculated mammary glands at 1, 3, and 6 w p.i. (n = 3 at each time point for 4T1 and Py230 primary tumors and Matrigel®-only inoculated BALB/c- and C57BL/6-derived mammary glands). Hierarchical clustering was performed using Euclidean distance. [file Image_5.TIF]
